# Demographic and regional disparities in concurrent respiratory and renal failure mortality: a CDC WONDER analysis, 1999–2024

**DOI:** 10.1038/s41598-026-55835-9

**Published:** 2026-06-12

**Authors:** Iftikhar khan, Ibrahiem Azeem Ajaz, Asma Chaudhary, Mirha Imran Khan, Rida Khan, Fnu Urooba, Nadia Siddiqui, Waqas Ali, Arham Khalid, Muhammad Sarim Azad Khan, Hamza Asif, Ahmed Saleem, Syed Nosherwan Ali, Aisha Chaudhary, Nawras Fashafsheh, Shankar Biswas

**Affiliations:** 1 FMH College of Medicine and Dentistry, Lahore, Pakistan; 2 Department of Medicine, Rashid Latif Medical College, Lahore, Pakistan; 3 Department of Medicine, Fazaia Medical College, Islamabad, Pakistan; 4 Combined Military Hospital Institute of Medical Science, Multan, Pakistan; 5 Combined Military Hospital Multan Institute of Medical Science, Multan, Pakistan; 6 Combined Military Hospital Institute of Medical Science, Multan, Pakistan; 7 HCA Florida Oakhill Hospital, Family Medicine, Florida, USA; 8 Lahore General Hospital, Lahore, Pakistan; 9 Rawalpindi Medical University, Rawalpindi, Pakistan; 10 Combined Military Hospital Lahore Medical College, Lahore, Pakistan; 11 University of Louisville Hospital, Kentucky, USA; 12 Department of Medicine, United Medical and Dental College, Karachi, Pakistan; 13 Combined Military Hospital Lahore Medical College, Lahore, Pakistan; 14 Department of Medicine, Fazaia Medical College, Islamabad, Pakistan; 15 Department of Nursing, Faculty of Health Sciences, Istanbul Gelisim University, Istanbul, Turkey; 16 Department of Internal Medicine, Ivano-Frankivsk National Medical University, Ivano-Frankivsk, Ukraine

**Keywords:** Respiratory failure, Renal failure, U.S. population, Mortality, Trends, Diseases, Health care, Medical research, Risk factors

## Abstract

Concurrent respiratory and renal failure carries exceptionally high mortality, yet population-level trends remain understudied. We analyzed temporal trends and demographic disparities in US mortality for this dual-organ failure from 1999 to 2024. We analyzed mortality data via CDC WONDER for deaths citing both respiratory (J96) and renal failure (N17–N19). Age-adjusted mortality rates (AAMRs) per 100,000 persons were extracted. Temporal trends were assessed using Joinpoint regression to estimate the Annual Percent Change (APC) with 95% confidence intervals (CIs). From 1999 to 2024, 894,269 US deaths involved concurrent respiratory and renal failure. AAMR increased from 5.76 to 14.01 per 100,000 population, rising from 1999 to 2011 (APC: 3.96), remaining stable during 2011–2018 (APC: −0.10; P = 0.90), sharply increasing during 2018–2021 (APC: 25.36), and declining from 2021 to 2024 (APC: −8.17). AAMR nearly doubled during 2020–2022 compared with 1999–2019 (16.37 vs. 8.06). Mortality was higher in men (12.01 vs. 7.66), non-Hispanic Blacks (15.42), and in the South and West, with non-metropolitan rates surpassing metropolitan areas in later years. Mortality from concurrent respiratory and renal failure more than doubled in the U.S. over the past two decades, with substantial disparities by sex, race/ethnicity, and geography. The sharp pandemic-era increase and persistent socio-regional inequities underscore a growing public health burden that warrants focused clinical attention and resource allocation.

**Supplementary Information:**

The online version contains supplementary material available at 10.1038/s41598-026-55835-9.

## Introduction

Respiratory failure represents a significant and escalating challenge to global healthcare systems, characterized by an immense clinical burden and surging hospitalizations, which increased by approximately 83% in the United States between 2002 and 2017^1^. While advances in diagnostic precision and ventilatory management have successfully reduced in-hospital mortality for isolated respiratory cases^1^, these survival gains are heavily overshadowed by the emergence of coexisting organ dysfunction^2,3^. The most critical and frequent complication driving high mortality in these patients is concurrent renal failure^2,3^. The co-occurrence of these conditions creates a lethal “therapeutic conflict”. Fluid resuscitation, essential for renal protection, can exacerbate pulmonary edema, while the conservative fluid strategies used to optimize oxygenation can precipitate or worsen kidney injury^4^. Because most evidence-based guidelines focus on single-organ failure, this combined phenotype lacks optimized protocols, leaving patients highly vulnerable.

Current literature describing this dual-organ burden originates almost exclusively from mechanistic studies and localized ICU-based clinical cohorts^2,3,5^. Relying solely on these highly controlled environments with limited sample sizes restricts the ability to extrapolate findings to population-level outcomes or identify systemic healthcare inequities. Furthermore, the individual economic toll of these conditions is astronomical: chronic kidney disease (CKD) alone accounted for over $87 billion in Medicare expenditures in 2019, while the addition of mechanical ventilation increases daily ICU costs by 25% to 30%^6,7^. Significant geographic and socioeconomic disparities are known to persist in the management of isolated organ failure^5,8–10^, but because current literature lacks a comprehensive national-level epidemiological characterization, it remains unknown how these structural vulnerabilities impact the mortality trajectory of populations facing concurrent respiratory and renal failure.

To address this critical epidemiological gap, we conducted a 25-year nationwide ecological analysis of mortality data from the National Vital Statistics System (NVSS) using the CDC WONDER online query system. Unlike clinical trials that focus on individual pathophysiology, this surveillance study aims to rigorously quantify the true population-level trajectory of concurrent respiratory and renal failure. By identifying long-term temporal trends, geographic disparities, and high-risk demographic subgroups, this analysis seeks to provide the foundational epidemiological data necessary to optimize resource distribution, guide targeted public health interventions, and address this compounding systemic crisis.

##  Methodology

### Study setting and population

We conducted a retrospective mortality analysis using death certificate data from 1999 to 2024 across all 50 U.S. states and the District of Columbia to assess mortality rates associated with concurrent respiratory and renal failure. Mortality data were obtained from the NVSS via the CDC WONDER online tool of the Centers for Disease Control and Prevention^11^. International Statistical Classification of Diseases and Related Health Problems-10th Revision (ICD-10) codes N17–N19 identified renal failure, and J96 identified respiratory failure, consistent with previous studies^12,13^. Using the ‘Multiple Cause of Death’ Public Use dataset, we included all cases where renal and respiratory failures appeared as either underlying or contributing causes of death^11^. To contextualize the mortality burden, we compared trends for concurrent failure against aggregate mortality trends for isolated respiratory failure (J96) and isolated renal failure (N17–N19). Sensitivity analyses stratified mortality by acute versus chronic pathology: respiratory failure with concurrent AKI (N17) versus with CKD (N18), and renal failure with acute (J96.0) versus chronic (J96.1) respiratory failure. The study followed STROBE (Strengthening the Reporting of Observational Studies in Epidemiology) guidelines^14^ and did not require IRB (institutional review board) approval because it used de-identified, publicly accessible data.

### Data abstraction

Mortality data were stratified by year, sex (male/female), race/ethnicity, and geographic markers, including states, census divisions, and urban-rural classifications. Age groups ranged from < 1 year to ≥ 85 years. Race and ethnicity were analyzed using the standard NCHS Bridged-Race categories provided within the CDC WONDER database, which harmonize racial classifications across the study period^15^. The categories included Hispanic or Latino, non-Hispanic (NH) White, NH Black or African American (AA), NH Asian or Pacific Islander, and NH American Indian or Alaska Natives (AI/AN). The place of death was categorized into medical facilities, home, hospice, or long-term care.

To map the longitudinal trajectory, the primary analysis covered 1999–2024. A comparative analysis for the stable pre-pandemic (1999–2019), pandemic period (2020–2022) used rate ratios (RR) and year-over-year percent changes to quantify the mortality surge. Urban-rural classification distinguished urban areas (large metropolitan areas over 1 million and medium/small metropolitan areas with 50,000–999,999 population) from rural areas (non-metropolitan areas under 50,000) per “The National Center for Health Statistics”^16^. The regions were categorized by the U.S. Census Bureau into Northeast, Midwest, South, and West^17^. Finally, in 2011, the NCHS updated the coding rules for renal failure, which artificially increased its selection as a cause of death. Readers should anticipate a corresponding administrative (non-epidemiological) discontinuity in the 2011–2012 data.

### Statistical analysis

Crude mortality rates (CMRs) and age-adjusted mortality rates (AAMRs) along with their 95% confidence intervals (CIs), were calculated per 100,000 population. CMRs were calculated as the annual number of deaths divided by the corresponding U.S. population, with AAMRs standardized to the 2000 U.S. population^18^. Temporal trends were assessed using the Joinpoint Regression Program (Version 6.0.1 National Cancer Institute) to estimate annual percent changes (APCs) via log-linear models^19^. APCs and their 95% CIs were derived for each segment between joinpoints using the “Monte Carlo permutation test” to identify the optimal number of inflection points, conducting 4,499 permutations^20^. Statistical significance of variations from baseline mortality was determined by the Joinpoint Regression Program (two-tailed t-tests), with p-values < 0.05 considered significant. Models were fit using the grid-search method assuming constant variance and uncorrelated errors. For stratified analyses (e.g., by sex or race), identical algorithm settings were applied, but models were fit independently without ensuring parallel trends or identical joinpoint locations, allowing the software to detect distinct temporal patterns for each demographic group.

## Results

From 1999 to 2024, 894,269 deaths involved concomitant renal and respiratory failure, predominantly in adults aged ≥ 65 years; children aged 1–4 years were least affected. Most deaths occurred in medical facilities, followed by nursing homes and decedent homes (Supplemental Table 1).

### Overall

**Fig. 1:**
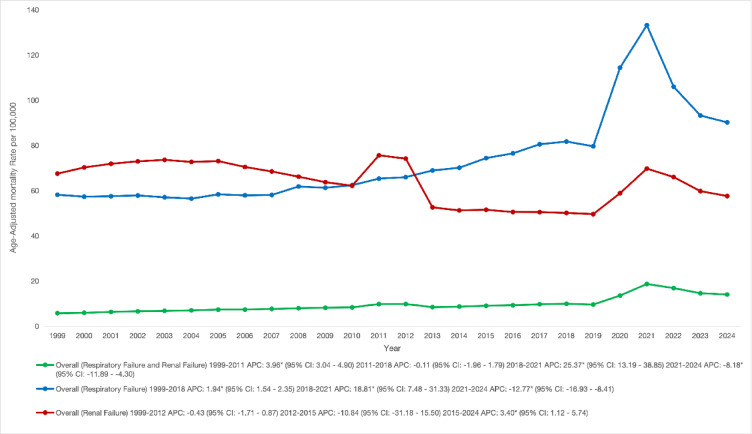
Age-adjusted mortality rates (AAMRs) per 100,000 for concurrent respiratory and renal failure, isolated respiratory failure, and isolated renal failure in the United States from 1999 to 2024. A dashed vertical line at 2011 denotes the National Center for Health Statistics (NCHS) coding transition period.

The AAMR for concomitant respiratory and renal failure increased from 5.76 in 1999 to 14.01 in 2024. AAMR increased from 1999 to 2011 (APC: 3.96; 95% CI: 3.03–4.90), remained stable during 2011–2018 (APC: −0.10; 95% CI: −1.96-1.79; *p* = 0.90), rose sharply from 2018 to 2021 (APC: 25.36; 95% CI: 13.19–38.85), then declined during 2021–2024 (APC: −8.17; 95% CI: −11.89 to −4.30) **(**Fig. 1; Table 1**).**

**Table 1:** Overall and sex-stratified concurrent respiratory & renal failure - related age-adjusted mortality rates per 100,000 in the United States, 1999 to 2024.

Year	Males AAMR (95% CI)	Females AAMR (95% CI)	Overall AAMR (95% CI)
1999	7.73 (7.57–7.90)	4.51 (4.40–4.61)	5.76 (5.67–5.85)
2000	7.96 (7.79–8.13)	4.67 (4.56–4.77)	5.94 (5.85–6.03)
2001	8.35 (8.18–8.52)	5.02 (4.91–5.12)	6.31 (6.22–6.41)
2002	8.65 (8.48–8.82)	5.26 (5.15–5.37)	6.57 (6.47–6.66)
2003	8.85 (8.67–9.02)	5.40 (5.29–5.52)	6.79 (6.70–6.89)
2004	9.08 (8.91–9.26)	5.63 (5.52–5.74)	7.00 (6.90–7.09)
2005	9.49 (9.31–9.67)	5.92 (5.81–6.04)	7.34 (7.24–7.43)
2006	9.43 (9.26–9.61)	5.97 (5.85–6.08)	7.35 (7.25–7.45)
2007	9.86 (9.69–10.03)	6.17 (6.06–6.29)	7.65 (7.55–7.74)
2008	10.10 (9.93–10.28)	6.48 (6.36–6.60)	7.94 (7.85–8.04)
2009	10.34 (10.17–10.52)	6.66 (6.54–6.78)	8.16 (8.07–8.26)
2010	10.52 (10.34–10.69)	6.79 (6.67–6.91)	8.33 (8.24–8.43)
2011	12.35 (12.17–12.54)	8.01 (7.88–8.14)	9.79 (9.69–9.90)
2012	12.37 (12.19–12.55)	7.93 (7.80–8.05)	9.81 (9.70–9.91)
2013	10.60 (10.43–10.77)	6.90 (6.78–7.01)	8.43 (8.34–8.53)
2014	10.85 (10.68–11.02)	7.08 (6.96–7.20)	8.67 (8.57–8.77)
2015	11.40 (11.23–11.57)	7.34 (7.22–7.46)	9.04 (8.94–9.14)
2016	11.65 (11.48–11.82)	7.44 (7.32–7.55)	9.26 (9.16–9.36)
2017	12.14 (11.97–12.31)	7.91 (7.79–8.03)	9.71 (9.61–9.81)
2018	12.44 (12.27–12.61)	8.06 (7.94–8.18)	9.93 (9.83–10.03)
2019	11.79 (11.63–11.95)	7.91 (7.80–8.03)	9.58 (9.49–9.68)
2020	17.55 (17.36–17.75)	10.48 (10.34–10.61)	13.55 (13.44–13.66)
2021	24.07 (23.84–24.31)	14.40 (14.24–14.56)	18.69 (18.55–18.82)
2022	21.33 (21.12–21.55)	13.50 (13.35–13.66)	16.86 (16.73–16.98)
2023	17.84 (17.65–18.04)	12.07 (11.93–12.21)	14.58 (14.46–14.69)
2024	17.06 (16.88–17.25)	11.64 (11.50–11.78)	14.01 (13.90–14.12)

### Stratified by sex

**Fig. 2:**
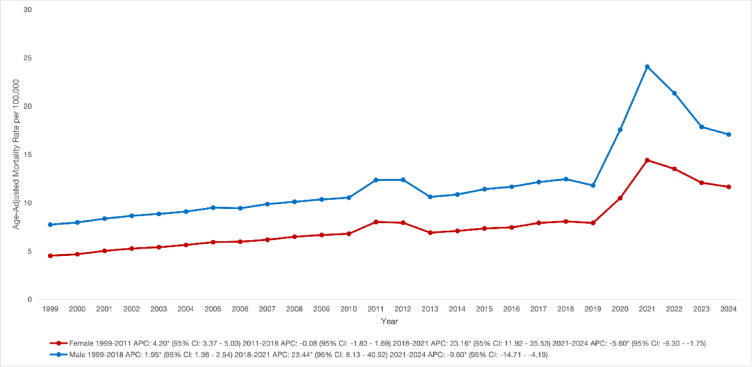
Age-adjusted mortality rates (AAMRs) per 100,000 for concurrent respiratory and renal failure stratified by sex in the United States, 1999–2024.

Males consistently had higher AAMRs than females (12.01 vs. 7.66). In males, AAMR increased from 1999 to 2018 (APC: 1.94; 95% CI: 1.36–2.54), rose sharply during 2018–2021 (APC: 23.44; 95% CI: 8.13–40.92), then declined from 2021 to 2024 (APC: −9.60; 95% CI: −14.71 to −4.19). In females, AAMR increased during 1999–2011 (APC: 4.19; 95% CI: 3.37–5.03), remained stable from 2011 to 2018 (APC: −0.08; 95% CI: −1.82-1.69; *p* = 0.92), increased sharply during 2018–2021 (APC: 23.16; 95% CI: 11.92–35.52), and declined from 2021 to 2024 (APC: −5.60; 95% CI: −9.29 to −1.75) **(**Fig. 2; Table 1**).**

### Stratified by race/ethnicity

**Fig. 3:**
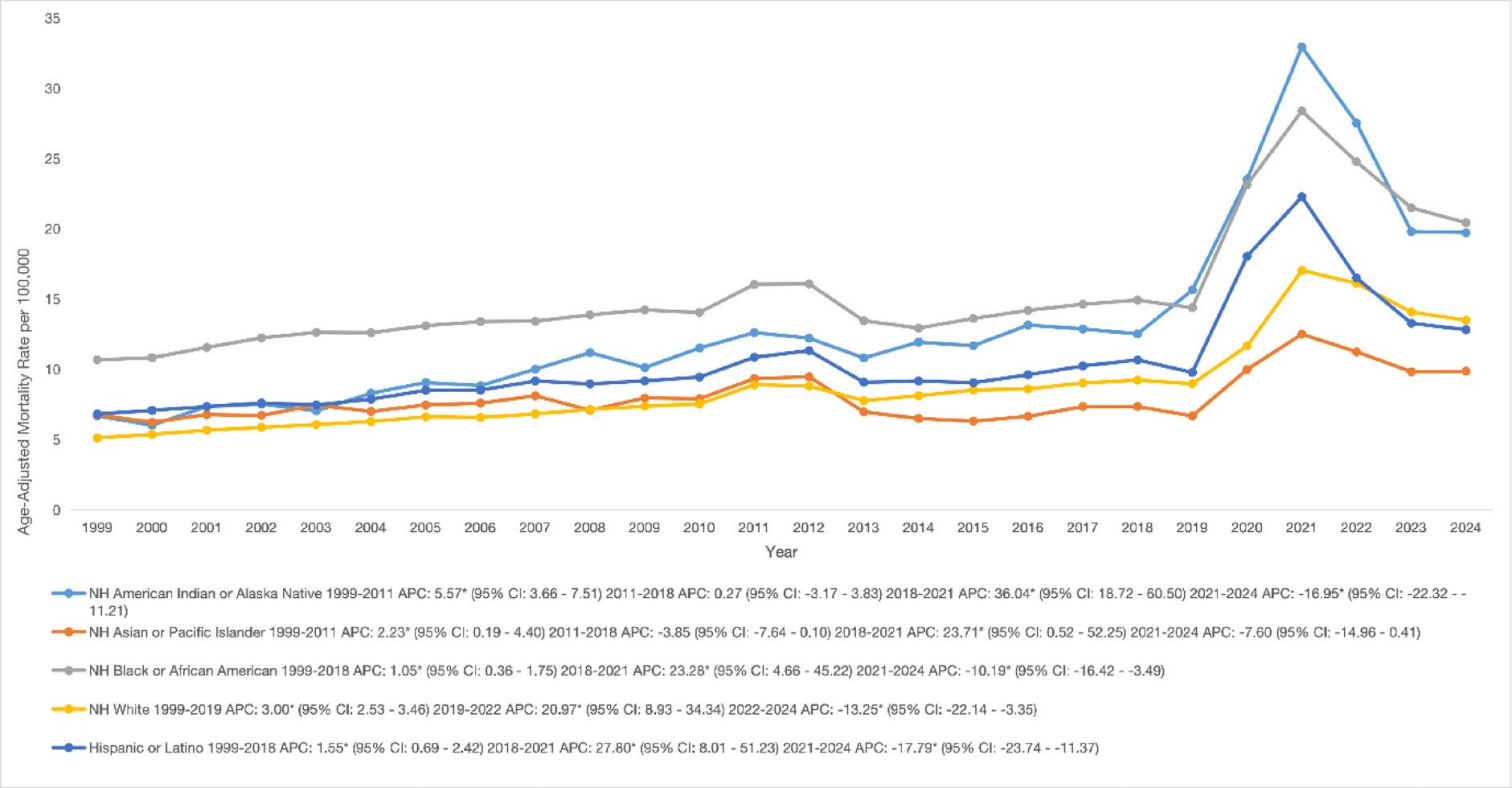
Age-adjusted mortality rates (AAMRs) per 100,000 for concurrent respiratory and renal failure stratified by race in the United States, 1999–2024.

By race/ethnicity, NH Black individuals had the highest overall AAMR (15.42), followed by NH AI/AN (13.09), Hispanic/Latino (10.40), NH White (8.71), and NH Asian/Pacific Islander (7.96). NH Whites showed increasing AAMRs during 1999–2019 (APC: 2.99; 95% CI: 2.53–3.46), a sharp rise in 2019–2022 (APC: 20.96; 95% CI: 8.92–34.33), then decline in 2022–2024 (APC: −13.25; 95% CI: −22.14 to −3.35). Mortality for NH Blacks and Hispanics increased during 1999–2018 (NH Black APC: 1.05; 95% CI: 0.35–1.74; Hispanic APC: 1.55; 95% CI: 0.70–2.41), rose sharply during 2018–2021 (NH Black APC: 23.28; 95% CI: 4.66–45.22; Hispanic APC: 27.80; 95% CI: 8.00–51.21), then declined during 2021–2024 (NH Black APC: −10.18; 95% CI: −16.42 to −3.49; Hispanic APC: −17.79; 95% CI: −23.74 to −11.36). NH AI/AN AAMR increased during 1999–2011 (APC: 5.56; 95% CI: 3.66–7.51), remained stable during 2011–2018 (APC: 0.27; 95% CI: −3.16 to 3.82; *p* = 0.87), increased sharply during 2018–2021 (APC: 38.03; 95% CI: 18.71–60.50), then declined during 2021–2024 (APC: −16.95; 95% CI: −22.31 to −11.21). NH Asian/Pacific Islanders increased during 1999–2011 (APC: 2.27; 95% CI: 0.18–4.40), remained stable during 2011–2018 (APC: −3.84; 95% CI: −7.64–0.10; *p* = 0.06), increased during 2018–2021 (APC: 23.71; 95% CI: 0.52–52.25), and remained stable during 2021–2024 (APC: −7.60; 95% CI: −14.96–0.41; *p* = 0.06) **(**Fig. 3; Table 2). Overall AAPCs were stable for NH Asians, NH Blacks, and Hispanics, but increased for NH Whites (3.58) and NH AI/ANs (4.40). Wide 2018–2021 CIs, particularly among Hispanics, NH Blacks, and NH AI/ANs, likely reflect pandemic-era death certification inconsistencies and should be interpreted cautiously.

**Table 2:** Concurrent respiratory & renal failure – related age-adjusted mortality rates per 100,000, stratified by race in the United States, 1999 to 2024.

Year	NH American Indian or Alaska Native	NH Asian or Pacific Islander	NH Black or African American	NH White	Hispanic or Latino
1999	6.67 (5.20–8.43)	6.74 (6.05–7.43)	10.67 (10.25–11.09)	5.11 (5.01–5.20)	6.81 (6.35–7.27)
2000	6.01 (4.68–7.61)	6.23 (5.59–6.88)	10.82 (10.40–11.24)	5.36 (5.26–5.45)	7.07 (6.61–7.54)
2001	7.34 (5.84–9.11)	6.77 (6.12–7.41)	11.55 (11.12–11.99)	5.66 (5.57–5.76)	7.34 (6.88–7.79)
2002	7.51 (5.97–9.04)	6.71 (6.09–7.33)	12.23 (11.78–12.67)	5.86 (5.77–5.96)	7.59 (7.14–8.04)
2003	7.03 (5.53–8.52)	7.44 (6.80–8.07)	12.62 (12.17–13.06)	6.06 (5.96–6.16)	7.46 (7.03–7.89)
2004	8.29 (6.69–9.88)	6.99 (6.39–7.58)	12.60 (12.16–13.04)	6.28 (6.18–6.38)	7.86 (7.43–8.29)
2005	9.05 (7.41–10.70)	7.46 (6.87–8.06)	13.10 (12.66–13.55)	6.61 (6.51–6.71)	8.50 (8.06–8.94)
2006	8.83 (7.29–10.37)	7.58 (7.00–8.17)	13.39 (12.95–13.84)	6.57 (6.46–6.67)	8.51 (8.09–8.94)
2007	10.00 (8.34–11.65)	8.11 (7.53–8.69)	13.42 (12.98–13.86)	6.82 (6.72–6.93)	9.16 (8.73–9.59)
2008	11.18 (9.48–12.87)	7.08 (6.55–7.60)	13.87 (13.42–14.31)	7.14 (7.04–7.25)	8.96 (8.54–9.37)
2009	10.11 (8.54–11.68)	7.95 (7.40–8.49)	14.22 (13.78–14.66)	7.36 (7.25–7.46)	9.17 (8.77–9.58)
2010	11.51 (9.83–13.19)	7.90 (7.36–8.43)	14.05 (13.61–14.48)	7.53 (7.42–7.63)	9.44 (9.03–9.84)
2011	12.61 (10.88–14.34)	9.33 (8.77–9.88)	16.04 (15.58–16.49)	8.89 (8.78–9.01)	10.85 (10.43–11.27)
2012	12.21 (10.58–13.84)	9.46 (8.92–10.00)	16.08 (15.63–16.53)	8.81 (8.70–8.93)	11.32 (10.90–11.73)
2013	10.80 (9.29–12.31)	6.96 (6.52–7.41)	13.45 (13.05–13.86)	7.76 (7.66–7.87)	9.07 (8.71–9.43)
2014	11.93 (10.40–13.46)	6.49 (6.08–6.90)	12.93 (12.54–13.32)	8.11 (8.00–8.21)	9.16 (8.81–9.51)
2015	11.67 (10.21–13.14)	6.31 (5.92–6.70)	13.61 (13.21–14.00)	8.52 (8.41–8.63)	9.04 (8.70–9.38)
2016	13.14 (11.60–14.68)	6.64 (6.25–7.03)	14.19 (13.79–14.59)	8.59 (8.48–8.70)	9.60 (9.26–9.94)
2017	12.87 (11.39–14.36)	7.34 (6.94–7.74)	14.63 (14.24–15.03)	9.02 (8.91–9.13)	10.23 (9.89–10.57)
2018	12.51 (11.09–13.93)	7.34 (6.95–7.72)	14.92 (14.53–15.31)	9.22 (9.11–9.33)	10.66 (10.32–11.00)
2019	15.64 (14.05–17.23)	6.67 (6.31–7.04)	14.38 (14.00–14.76)	8.97 (8.86–9.08)	9.77 (9.45–10.09)
2020	23.52 (21.62–25.41)	9.98 (9.56–10.41)	23.14 (22.66–23.61)	11.65 (11.53–11.78)	18.04 (17.62–18.46)
2021	32.95 (30.67–35.38)	12.49 (12.01–12.98)	28.39 (27.86–28.92)	17.03 (16.88–17.18)	22.28 (21.82–22.74)
2022	27.52 (25.47–29.70)	11.24 (10.80–11.70)	24.77 (24.28–25.26)	16.12 (15.98–16.27)	16.50 (16.11–16.89)
2023	19.80 (18.09–21.66)	9.82 (9.41–10.23)	21.49 (21.04–21.95)	14.07 (13.93–14.21)	13.27 (12.93–13.62)
2024	19.72 (18.05–21.53)	9.85 (9.46–10.26)	20.43 (20.00–20.87)	13.49 (13.36–13.63)	12.81 (12.48–13.14)

### Stratified by geographic regions

**Fig. 4:**
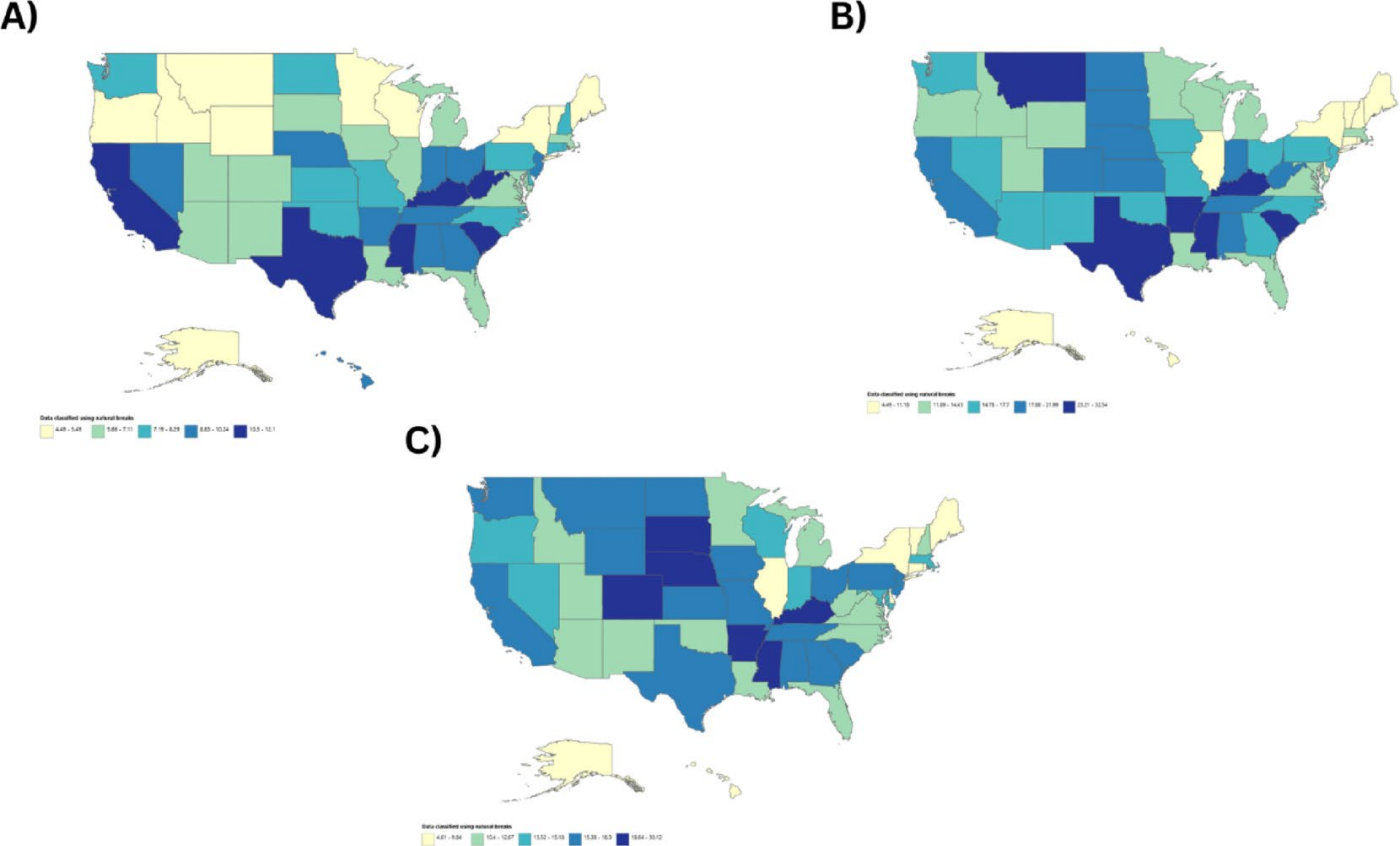
Geographic distribution of age-adjusted mortality rates (AAMRs) per 100,000 population for concurrent respiratory and renal failure across U.S. states during (A) 1999–2019, (B) 2020–2022, and (C) 2023–2024. State-level rates were classified using natural break (Jenks) intervals.

Pre-COVID (1999–2019), state AAMRs ranged from 12.21 in Texas to 4.49 in Oregon; Mississippi, South Carolina, and West Virginia ranked in the upper 90th percentile, while Alaska, Minnesota, Virginia, Wisconsin, and Wyoming were in the lowest 10th percentile **(**Fig. 4A**).** During COVID-19 (2020–2022), Arkansas, Montana, South Carolina, Mississippi, and Kentucky had the highest AAMRs, whereas Maine, Vermont, New York, and Hawaii had the lowest **(**Fig. 4B**)** Post-COVID (2023–2024) AAMR was highest for Colorado, Nebraska, Mississippi, Arkansas, and Kentucky while lowest for Maine, Rhode Island, Connecticut, and Vermont (Fig.4C, Supplemental Table 2).

Regionally, the South had the highest overall AAMR (9.48), followed by the West (9.07), the Midwest (8.63), and the Northeast (8.08). AAMR increased during 1999–2018 in the South (APC: 2.81; 95% CI: 2.26–3.37) and Midwest (APC: 2.65; 95% CI: 2.10–3.20), rose sharply during 2018–2021 (South APC: 21.44; 95% CI: 6.96–37.89; Midwest APC: 21.57; 95% CI: 6.03–39.58), then declined during 2021–2024 (South APC: −9.65; 95% CI: −14.56 to −4.47; Midwest APC: −5.48; 95% CI: −10.60 to −0.05). In the Northeast, AAMR increased during 1999–2011 (APC: 2.56; 95% CI: 1.69–3.42), remained stable during 2011–2018 (APC: −1.55; 95% CI: −3.50-0.44; *p* = 0.11), surged during 2018–2021 (APC: 24.38; 95% CI: 12.14–37.95), then declined during 2021–2024 (APC: −6.33; 95% CI: −10.48 to −1.98). Mortality in the West increased during 1999–2011 (APC: 3.81; 95% CI: 2.61–5.04), remained stable during 2011–2018 (APC: −0.65; 95% CI: −3.14 to 1.90; *p* = 0.59), increased during 2018–2021 (APC: 27.15; 95% CI: 11.37–45.19), then declined during 2021–2024 (APC: −7.8; 95% CI: −12.72 to −2.63) (Supplemental Table 3).

**Fig. 5:**
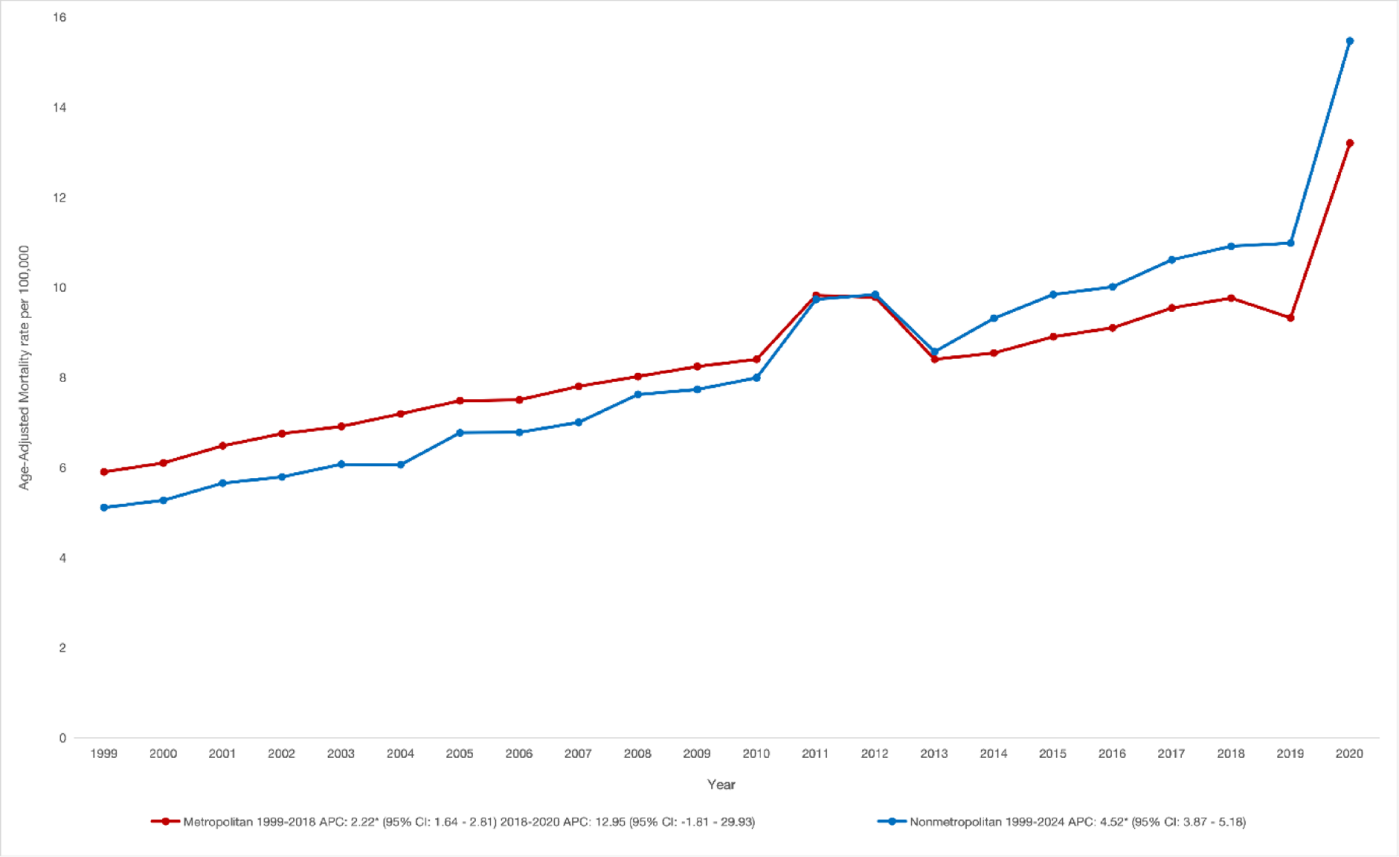
Age-adjusted mortality rates (AAMRs) per 100,000 for concurrent respiratory and renal failure stratified by urban–rural status in the United States, 1999–2020.

From 1999 to 2020, metropolitan and non-metropolitan AAMRs were similar (8.50 vs. 8.50), but non-metropolitan mortality increased more rapidly, surpassed metropolitan rates by 2013, and peaked higher during 2020 (15.47 vs. 13.20) (Fig.5, Supplemental Table 4).

### Comparison with individual organ failures

Respiratory failure had an overall AAMR of 73.2, increasing during 1999–2018 (APC: 1.94), rising sharply during 2018–2021 (APC: 18.80), then declining during 2021–2024 (APC: −12.77). Renal failure had an overall AAMR of 62.53 and showed non-significant declines during 1999–2012 (APC: −0.42; *p* = 0.49) and 2012–2015 (APC: −10.8; *p* = 0.36), followed by an increase during 2015–2024 (APC: 3.40) (Fig.1, Supplemental Table 5).

### Sensitivity analysis by organ failure phenotype

#### Sensitivity analysis by renal phenotype

**Fig. 6:**
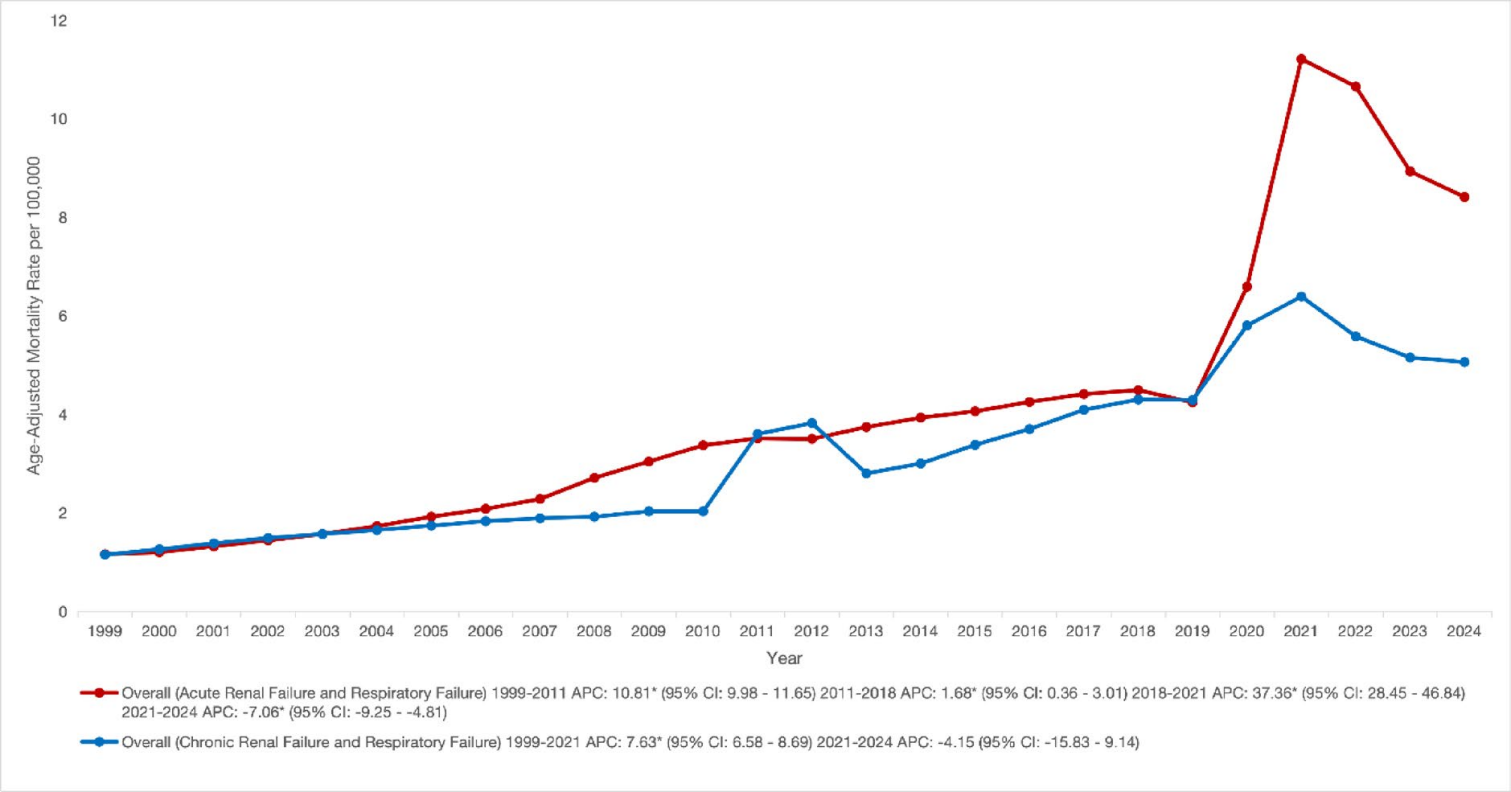
Age-adjusted mortality rates (AAMRs) per 100,000 for concurrent respiratory and renal failure stratified by acute versus chronic renal failure in the United States, 1999–2024.

Mortality associated with acute renal failure and concomitant respiratory failure increased during 1999–2011 (APC: 10.81; 95% CI: 9.98–11.65), continued rising during 2011–2018 (APC: 1.68; 95% CI: 0.36–3.01), surged during 2018–2021 (APC: 37.36; 95% CI: 28.49–46.84), then declined during 2021–2024 (APC: −7.06; 95% CI: −9.25 to −4.80). CKD with respiratory failure showed a sustained increase during 1999–2021 (APC: 7.62; 95% CI: 6.58–8.68). From 2021 to 2024, AAMR remained stable (APC: −4.15; 95% CI: −15.82-9.14, *p* = 0.5) (Fig. 6, Supplemental Table 6).

#### Sensitivity analysis by respiratory phenotype

**Fig. 7:**
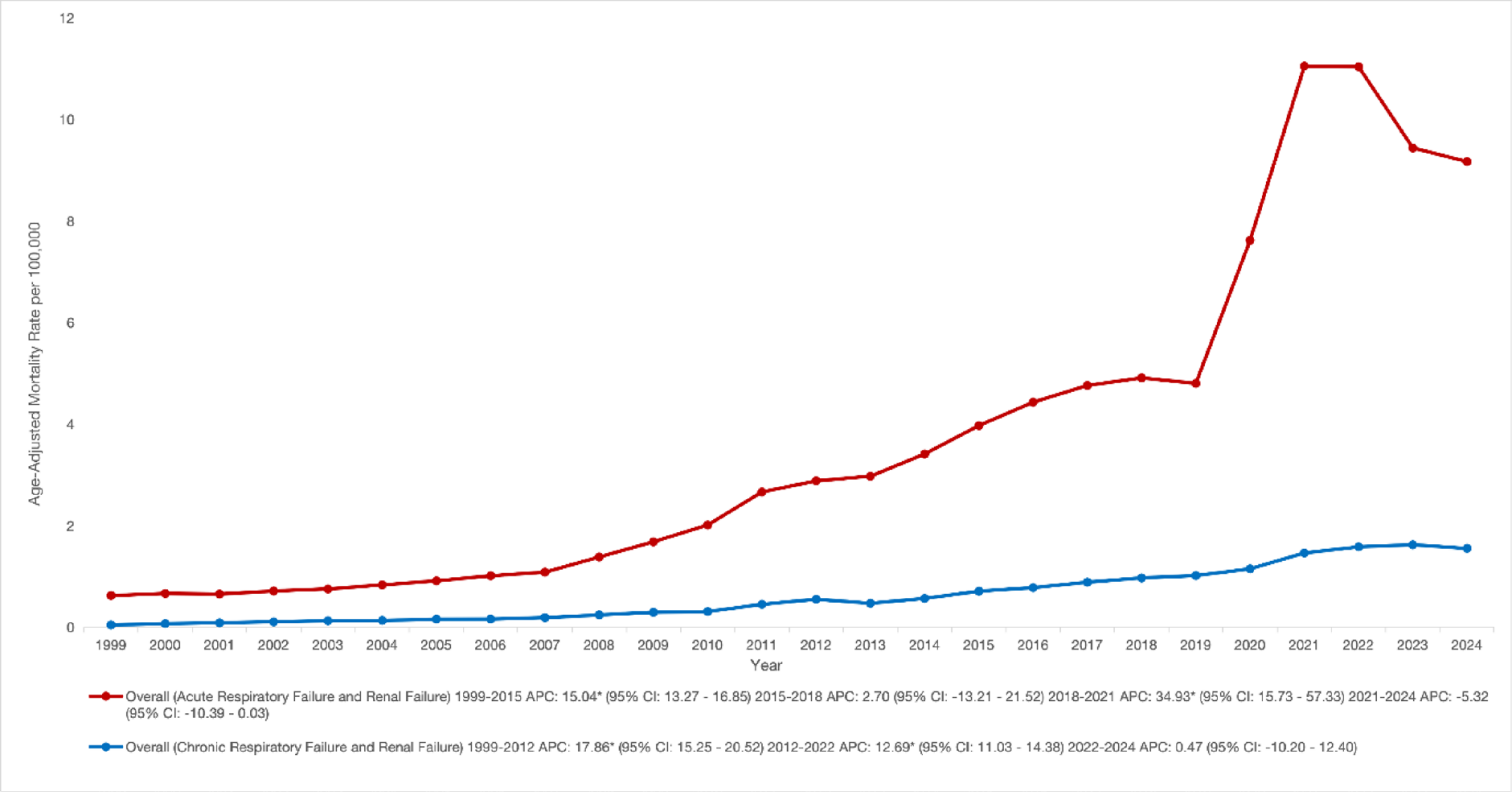
Age-adjusted mortality rates (AAMRs) per 100,000 for concurrent respiratory and renal failure stratified by acute versus chronic respiratory failure in the United States, 1999–2024.

Acute respiratory failure with concomitant renal failure showed a rapid increase in mortality from 1999 to 2015 (APC: 15.04; 95% CI: 13.27–16.84), stabilization from 2015 to 2018 (APC: 2.69; 95% CI: −13.20–21.51; *p* = 0.74), a sharp rise from 2018 to 2021 (APC: 34.93; 95% CI: 15.72–57.32), and stabilization from 2021 to 2024 (APC: 95% CI: −5.3; 95% CI: −10.38 to 0.025; *p* = 0.05). Chronic respiratory failure with renal failure showed a sustained increase from 1999 to 2012 (APC: 17.86; 95% CI: 15.25–20.52), continued rise from 2012 to 2022 (APC: 12.69; 95% CI: 11.03–14.37), and stabilization from 2022 to 2024 (APC: 0.46; 95% CI: −10.20 to 12.40; *p* = 0.93) **(**Fig.7, Supplementary Table 7).

### Stratified by pre–pandemic (1999–2019) and pandemic period (2020–2022)

The average AAMR during the pandemic period (2020–2022) was 16.37, which was 2.03 times higher than the pre-pandemic average of 8.06 (Rate Ratio: 2.03). Examining the immediate impact, mortality surged by 41.4% from 2019 (AAMR: 9.58) to 2020 (AAMR: 13.55). This upward trajectory continued with a further 37.9% increase from 2020 to 2021 (AAMR: 18.69), followed by a 9.8% decrease in 2022 (AAMR: 16.86) **(**Fig. 1; Table 1**)**.

### Distribution of underlying cause of death (UCOD) among decedents with concurrent respiratory and renal failure, pre-pandemic, pandemic, post-pandemic

Pre-pandemic (1999–2019), renal failure was the UCOD in 20.64% of deaths and respiratory failure in 0.77%, while 78.59% were due to other causes (e.g., sepsis, cardiovascular disease, malignancy). Stratified analysis showed higher attribution in chronic phenotypes: renal failure in 25.51% of CKD cases and respiratory failure in 8.60% of chronic respiratory failure cases. During 2020–2022, among 202,297 decedents with concurrent respiratory and renal failure, COVID-19 was the leading UCOD (27.3%; *N* = 55,228), followed by renal failure (14.2%; *N* = 28,709) and malignancies (7.6%; *N* = 15,379). Post-pandemic (2023–2024), renal failure accounted for 16.72% of UCOD, while respiratory failure accounted for 1.04% (Supplemental Table 8).

## Discussion

**Fig. 8:**
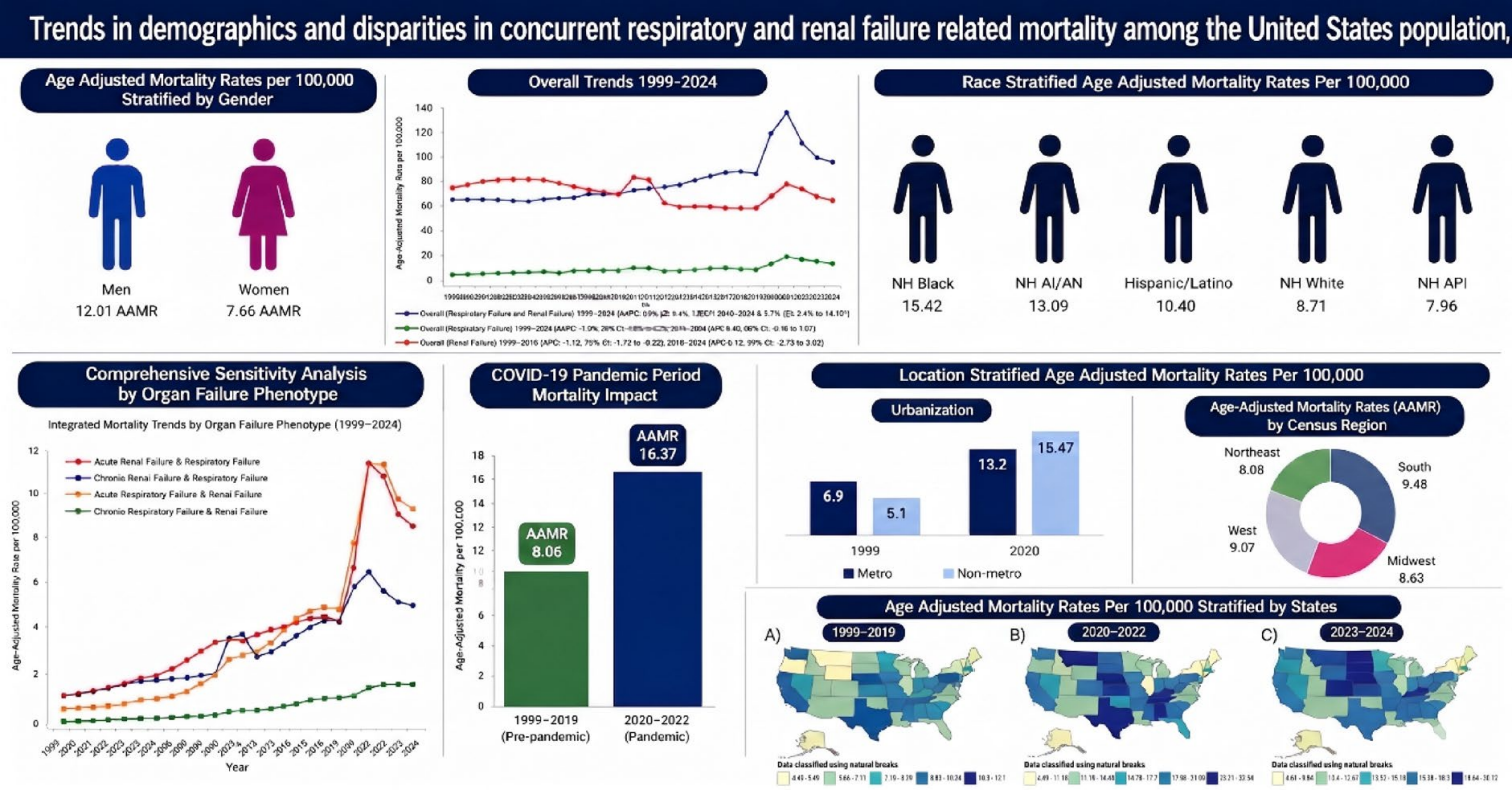
Central illustration, trends in demographics and disparities in concurrent respiratory and renal failure-related mortality among the United States population, 1999–2024.

This nationwide CDC WONDER analysis demonstrates a substantial and sustained increase in mortality associated with concomitant respiratory and renal failure in the United States from 1999 to 2024. During this study period, the overall AAMR more than doubled, marked by a gradual rise prior to 2011, relative stabilization from 2011 to 2018, a sharp surge between 2018 and 2021, and a partial decline thereafter. This mortality burden disproportionately impacted older adults, males, individuals residing in institutional healthcare settings, and NH AA, whereas NH Asians consistently exhibited the lowest rates across all age groups. Furthermore, pronounced regional inequities emerged, with the Southern region particularly Mississippi, South Carolina, and West Virginia bearing the highest mortality, in stark contrast to the lowest rates observed in states like Alaska, Minnesota, Virginia, Wisconsin, and Wyoming. Lastly, the COVID-19 pandemic precipitated a profound mortality shock, significantly exacerbating this trend by doubling the average AAMR from a pre-pandemic baseline of 8.06 to 16.37 during the 2020–2022 period. **(**Fig. 8**)**

Our findings highlight concurrent respiratory and renal failure as an escalating clinical challenge with a severe mortality trajectory. While our population-level data preclude causal inference, this trend aligns with the recognized pathophysiological complexities of lung–kidney crosstalk^2–4^. Specifically, AKI promotes volume overload, systemic inflammation, pulmonary edema, and acute lung injury, while uremic toxins increase pulmonary capillary permeability and thus can contribute to “uremic lung”^4^. Conversely, acute respiratory failure and positive-pressure ventilation may impair renal perfusion through hemodynamic instability, systemic inflammation, and elevated central venous pressure, precipitating secondary kidney injury^2–4^. Beyond population aging, the rising mortality burden is likely influenced by the two-decade increase in cardio-metabolic risk factors, including obesity, metabolic syndrome, and type 2 diabetes. These conditions increase the prevalence of baseline CKD^21^, thereby potentially leaving a growing proportion of patients with limited renal reserve to withstand the compounding physiological stress of acute respiratory illness.

The plateau in mortality between 2011 and 2018 suggests stabilization driven by advancements in critical care and chronic disease management^1^ such as sepsis management bundles, lung-protective ventilation^9^ and standardized AKI/CKD guidelines, including KDIGO^1,3,4,21–23^. In contrast, the marked mortality surge during 2018–2021 likely reflects the profound impact of the COVID-19 pandemic. Severe acute respiratory syndrome coronavirus 2 (SARS-CoV-2) primarily affects the respiratory system but is also strongly associated with AKI and systemic inflammation. Cytokine storm, endothelial dysfunction, mechanical ventilation, and hemodynamic instability have been proposed as potential contributors to concurrent pulmonary and renal failure in critically ill patients^24^. Prior studies have shown that AKI among hospitalized COVID-19 patients is associated with substantially increased mortality, particularly in mechanically ventilated individuals^25^. Beyond direct viral effects, healthcare system strain during pandemic peaks including emergency department overcrowding, shortages of ventilators, continuous renal replacement therapy (CRRT) resources, and nephrology personnel, likely further worsened outcomes^26^. Because ICU strain is an independent driver of poor patient outcomes, the observed spike in AAMR is hypothesized to represent ‘resource-limited mortality’, in which mortality increased due to the unavailability of specialized interventions rather than disease severity alone^26^. These findings underscore the need for scalable and equitable resource-allocation strategies during healthcare crises. Notably, evidence from the COVID-19 pandemic suggests “Medicaid expansion” was associated with smaller increases in all-cause mortality^27^, warranting future investigation into whether similar policy mechanisms could mitigate mortality related to concurrent organ failure in vulnerable populations.

The post-2021 mortality decline aligns with mass vaccination campaigns^28^, rising population immunity, milder variants (e.g., Omicron)^29^, and optimized therapeutics (e.g., antivirals, immunomodulators)^30^. However, rates remain substantially higher than early-2000s baselines. This persistent excess mortality suggests the pandemic did not merely act as a transient shock, but rather amplified preexisting systemic vulnerabilities, compounded by long-term COVID-19 sequelae and ongoing healthcare disparities^31^.

We stratified mortality trends by both renal and respiratory phenotypes. Our analysis revealed sharp mortality increases for respiratory failure concurrent with both AKI and CKD. This dual trajectory suggests that the observed mortality burden may not be driven solely by one phenotype but reflects a compounding crisis of both acute and chronic disease progression. Additionally, a significant mortality discontinuity observed between 2010 and 2012 coincides with 2011 NCHS coding updates, which artificially increased the selection of kidney disease as an underlying cause of death by 13.5%^32^. While this administrative artifact caused local volatility, the overarching 26-year upward trajectory remains robust and seems independent of this classification shift.

Males exhibited higher overall mortality, consistent with greater biological susceptibility (e.g., higher ACE2 levels, hormonal effects, sex-dependent immunity)^33,34^, behavioral risks (e.g., smoking, occupational exposures, delayed healthcare)^35^, and a higher prevalence of cardiometabolic comorbidities (hypertension, diabetes mellitus, and CVD)^36^. Conversely, female hormones confer protective effects that mitigate CKD progression^37^ and generally have more robust immune responses and higher resistance to infection^4,21,38^. Despite lower overall mortality, females demonstrated distinct phases: an initial rise (1999–2011), a plateau (2011–2018), a sharp spike (2018–2021), and a subsequent decline through 2024. The early increase likely reflects demographic shifts such as increased longevity and improved survival from cardiovascular disease^33,34^, rising obesity, and a growing CKD burden in older females^21,37^, compounded by care disparities including healthcare access, diagnostic thresholds, disease recognition^39,40^ and under-prescription of guideline-directed therapies^41^. The 2011–2018 plateau coincided with national public health initiatives focused on cardiovascular health and symptom recognition in women. Improved incorporation of sex-specific data into prevention strategies and better hypertension control may have helped slow the rise in mortality trends^42^. The rapid 2018–2021 surge may likely be driven by COVID-19 exacerbating baseline comorbidities^43,44^. Ultimately, these distinct trajectories emphasize the critical need for enhanced, sex-specific risk stratification and interventions.

In our study, NH Black individuals experienced the highest overall mortality, followed by NH AI/AN populations, whereas NH Asian/Pacific Islanders demonstrated the lowest mortality rates^45^. The elevated burden among NH Black populations was potentially driven by the earlier onset of hypertension and obesity^46^, accelerated progression to CKD^47^, and socioeconomic disadvantages contributing to delayed nephrology referral, poorer dialysis adherence, higher dialysis-related mortality, and lower utilization of arteriovenous fistulas and peritoneal dialysis due to inadequate patient support and communication barriers^48,49^. NH Blacks also experience disproportionately higher mortality from respiratory diseases, likely related to greater exposure to environmental pollutants and the cumulative effects of socioeconomic disadvantage^50^. NH AI/AN populations demonstrated particularly concerning mortality trajectories, including the highest overall AAPC and the steepest pandemic-era increase. These findings are especially important given the persistent underrepresentation of Indigenous populations in national epidemiologic analyses despite a disproportionately high chronic disease burden^51^. Disparities in this group appear to be driven primarily by healthcare infrastructure limitations and geographic isolation. Chronic underfunding of healthcare systems^52^, shortages of specialty services, transportation barriers, and delayed emergency response capacity likely contributed to worse outcomes in severe respiratory and renal illness, compounded by rural hospital closures that severely restrict access to dialysis and intensive care^53–55^. During 2018–2021, pandemic-era vulnerabilities, including overcrowded housing and reduced preventive care capacity, may have intensified mortality in these communities^56^.

Conversely, NH Asian/Pacific Islanders demonstrated the lowest overall mortality rates and relatively stable long-term trends. Potential contributors may include lower smoking prevalence in some Asian subgroups, more favorable cardiovascular risk profiles, stronger community-level social support, and lower baseline prevalence of severe chronic pulmonary disease^13,57^. However, interpretation should be cautious because the NH Asian/Pacific Islander category encompasses highly heterogeneous populations with substantial differences in socioeconomic status, immigration history, healthcare access, and disease burden. Aggregation of these populations within national datasets may obscure important subgroup disparities^58^. Despite overall lower mortality, a sharp rise during the pandemic was observed, likely contributing to underreported infections, limited testing access, and delayed hospital presentation in minority communities^59,60^. Prior studies also reported longer symptom-to-admission intervals and higher rates of critical illness, mechanical ventilation, and ICU admission among Asian Americans^61,62^.

Significant geographic disparities, particularly the high mortality burden in the South, may reflect potential ecological correlates such as poor healthcare capacity, socioeconomic marginalization, and regional cultural factors impacting disease care and outcomes^63,64^. Notably, the steeper trajectory and acceleration of mortality in non-metropolitan areas in early 2013 temporally align with two major structural shifts in US healthcare. First, while an initial wave of rural hospital closures began around 2010, this systemic loss of infrastructure reached an unprecedented, sharp acceleration after 2013^53^. This compounding loss of local resources is hypothesized to have increased emergency transport times and restricted timely access to critical care stabilization^54^. For vulnerable rural populations experiencing rapid respiratory and renal decline, these structural delays in receiving advanced organ support may have been independently associated with significantly increased inpatient mortality^55^. Second, while the 2014 implementation of the Affordable Care Act (ACA) Medicaid expansion improved outcomes nationally, many states with the highest rural and Southern populations opted out. This exacerbated coverage gaps and accelerated financial distress for rural safety-net hospitals^65^, which may have served as a compounding systemic pressure, potentially contributing to delayed care until their organ failure was acute, irreversible, and ultimately fatal. However, it is critical to note that these structural vulnerabilities remain speculative ecological correlates, and direct individual-level causal links cannot be established from this aggregate data. And so for healthcare systems, particularly in the Southern United States and rural areas where mortality is highest, our results support the implementation of early bidirectional screening protocols; for instance, the presence of acute respiratory distress should trigger immediate and frequent monitoring of renal biomarkers to forestall the compounded risk of dual-organ failure.

The concentration of deaths in medical facilities (followed by nursing homes) highlights the acute, critical nature of combined respiratory and renal failure. Management of this condition inherently requires high-resource interventions, such as invasive mechanical ventilation and CRRT^5^. The high proportion of facility-based deaths emphasizes the immense strain this specific phenotype of multi-organ failure places on ICU resources and healthcare infrastructure^7,9^.

Crucially, the concurrent presentation of respiratory and renal failure must be conceptualized within the broader cardiovascular axis. In clinical practice, this dual-organ dysfunction frequently manifests as a downstream consequence of primary CVD, particularly acute decompensated heart failure and cardiorenal syndromes. Elevated cardiac filling pressures and pulmonary venous congestion may precipitate pulmonary edema and respiratory failure, while reduced cardiac output, venous congestion, systemic inflammation, and neurohormonal activation simultaneously impair renal perfusion and accelerate kidney injury^66^. This mechanistic link is further supported by our UCOD findings, which identify CVD as the predominant primary etiology, rather than isolated pulmonary or intrinsic renal pathology. Consequently, the lethal ‘therapeutic conflict’ of fluid resuscitation is ultimately a triad of cardiopulmonary-renal interactions. Paradoxically, longitudinal advancements in acute cardiovascular care have likely contributed to these rising mortality trends; as more patients survive acute myocardial infarctions, the population living with chronic heart failure and progressive CKD has expanded, creating a vastly larger at-risk pool^21^. Mitigating this mortality burden requires a paradigm shift from reactive, downstream organ support (e.g., mechanical ventilation and dialysis) to the proactive, upstream management of systemic antecedents, principally CVD, sepsis, and metabolic dysregulation. Finally, managing this highly complex patient population necessitates a reevaluation of structural healthcare policies. Initiatives such as the Hospital Readmissions Reduction Program (HRRP) warrant ongoing scrutiny to ensure they do not inadvertently incentivize the premature discharge of medically fragile patients, uniquely vulnerable to rapid cardio-renal decompensation^67^.

### Limitations

This study has several limitations. First, ICD-10 co-listing captures concurrent syndromic burden but cannot determine the temporal sequence of organ dysfunction. Second, the aggregate nature of the data precluded adjustment for individual-level clinical and socioeconomic confounders, although geographic and racial stratifications served as structural proxies. Third, death certificate analyses are inherently vulnerable to misclassification bias. Racial misclassification, particularly among AI/AN, Asian/Pacific Islander, and Hispanic populations, may underestimate disparities^68^. Respiratory failure is often coded as a terminal physiologic event rather than a primary pathology, and temporal changes in ICD-10 reporting and critical care coding practices may have influenced observed mortality trends^69,70^. Variable physician reporting accuracy and underreporting of chronic comorbidities may introduce additional bias^71^. Fourth, use of NCHS Bridged-Race estimates could’ve resulted in minor misclassification in multiracial populations. Fifth, overlap between comparative “single-cause” and concurrent cohorts prevented formal interaction analyses to confirm biological synergy. Sixth, pandemic-era APC estimates should be interpreted cautiously, particularly in smaller racial/ethnic groups with wide CIs, as 2018–2021 trends likely reflect acute COVID-19-related mortality shocks rather than stable secular changes. However, inclusion of post-pandemic data through 2024 enabled evaluation of subsequent recovery trajectories, supplemented by separate pre-pandemic and pandemic-period rate ratio analyses. Despite these limitations, this nationwide analysis provides important epidemiological insights for resource allocation and prevention in vulnerable populations.

## Conclusions

Mortality from concurrent respiratory and renal failure has increased substantially in the U.S., exceeding trends observed with isolated organ failure. The burden disproportionately affected older adults, males, NH Black populations, South and rural communities, and was markedly amplified during the COVID-19 pandemic, highlighting vulnerability to systemic healthcare strain. Most deaths occurred in medical facilities, suggesting acute terminal decompensation rather than expected palliative progression. These findings underscore a growing public health challenge and identify concurrent respiratory and renal dysfunction as an important target for future epidemiological and clinical research.

## Supplementary Information

Below is the link to the electronic supplementary material.

Supplementary Material 1

## Data Availability

The datasets used for analysis and interpretation were obtained from the CDC WONDER Multiple Cause of Death database, which are available publicly on the CDC website.

## Abbreviations

AAMR: Age-adjusted mortality rate
APC: Annual percent change
ARF: Acute respiratory failure
AKI: Acute kidney injury
ARDS: Acute respiratory distress syndrome
CDC WONDER: Centers for disease control and prevention wide-ranging online data for epidemiologic research
CI: Confidence intervals
CKD: Chronic kidney disease
CMR: Crude mortality rates
COPD: Chronic obstructive pulmonary disease
GFR: Glomerular filtration rate
ICD: International statistical classification of diseases and related health problems
NH: non-hispanic
STROBE: Strengthening the reporting of observational studies in epidemiology
