# Supplementary material for: Demographic and regional disparities in concurrent respiratory and renal failure mortality: a CDC WONDER analysis, 1999–2024
Source: Sci Rep. 2026 Jun 12;16:24515. doi: 10.1038/s41598-026-55835-9 (PMC13451193; doi:10.1038/s41598-026-55835-9)
Supplement: Supplementary file 1 [file 41598_2026_55835_MOESM1_ESM.docx]

**SUPPLEMENTAL TABLES**

**Supplemental Table 1: Concurrent Respiratory & Renal Failure, Mortality Stratified by Place of Death in the United States, 1999 to 2024**

**Supplemental Table 2: Concurrent Respiratory & Renal Failure – Related Age-Adjusted Mortality Rates per 100,000, Stratified by States in the United States, 1999 to 2024**

**Supplemental Table 3: Concurrent Respiratory & Renal Failure – Related Age-Adjusted Mortality Rates per 100,000, Stratified by Census Region in the United States, 1999 to 2024**

**Supplemental Table 4: Concurrent Respiratory & Renal Failure – Related Age-Adjusted Mortality Rates per 100,000, Stratified by Urban-Rural Classification in the United States, 1999 to 2024**

**Supplemental Table 5: Respiratory, Renal and Heart Failure – Related Age-Adjusted Mortality Rates per 100,000, in the United States, 1999 to 2024**

**Supplemental Table 6: Concurrent Respiratory & Renal Failure - Related Age-Adjusted Mortality Rates per 100,000 Stratified by Renal Failure Subtype (Acute vs Chronic Renal Failure) in the United States, 1999–2024**

**Supplemental Table 7: Concurrent Respiratory & Renal Failure - Related Age-Adjusted Mortality Rates per 100,000 Stratified by Respiratory Failure Subtype (Acute vs Chronic Respiratory Failure) in the United States, 1999–2024**

**Supplemental Table 8: Distribution of Underlying Cause of Death Among Decedents With Concurrent Respiratory and Renal Failure, Stratified by Renal and Respiratory Phenotypes, in the United States**

**Supplemental Table 1: Concurrent Respiratory & Renal Failure, Mortality Stratified by Place of Death in the United States, 1999 to 2024**

| **Year** | **Medical Facility** | **Nursing Home / LTC** | **Hospice** | **Home** | **Place of Death Unknown** | **Other** |
| --- | --- | --- | --- | --- | --- | --- |
| 1999 | 13607 | 1145 | — | 460 | — | 113 |
| 2000 | 14017 | 1252 | — | 556 | — | 132 |
| 2001 | 15118 | 1442 | — | 611 | — | 159 |
| 2002 | 15896 | 1463 | — | 641 | — | 224 |
| 2003 | 16693 | 1533 | 15 | 725 | 82 | 205 |
| 2004 | 17292 | 1613 | 19 | 829 | 88 | 271 |
| 2005 | 18532 | 1725 | 94 | 836 | 79 | 269 |
| 2006 | 18908 | 1674 | 172 | 898 | 59 | 269 |
| 2007 | 19998 | 1796 | 310 | 994 | 31 | 236 |
| 2008 | 21002 | 1734 | 389 | 990 | 432 | 228 |
| 2009 | 21600 | 1807 | 460 | 998 | 747 | 273 |
| 2010 | 22818 | 1846 | 680 | 1189 | 20 | 266 |
| 2011 | 26741 | 2524 | 961 | 1727 | 24 | 444 |
| 2012 | 26997 | 2640 | 1199 | 1856 | 17 | 501 |
| 2013 | 24761 | 1862 | 913 | 1357 | 22 | 458 |
| 2014 | 25756 | 2072 | 1145 | 1486 | 21 | 358 |
| 2015 | 27587 | 2215 | 1263 | 1618 | 15 | 303 |
| 2016 | 28429 | 2229 | 1431 | 1913 | 15 | 334 |
| 2017 | 29997 | 2525 | 1774 | 2185 | 7 | 399 |
| 2018 | 31367 | 2606 | 1928 | 2402 | — | 358 |
| 2019 | 30598 | 2626 | 1932 | 2370 | — | 362 |
| 2020 | 44995 | 3396 | 2275 | 3440 | 11 | 559 |
| 2021 | 64342 | 2951 | 2457 | 3731 | 26 | 745 |
| 2022 | 59758 | 3059 | 2331 | 3495 | 19 | 760 |
| 2023 | 51191 | 3105 | 2230 | 3376 | 16 | 576 |
| 2024 | 50173 | 3112 | 2375 | 3484 | 20 | 630 |

**Supplemental Table 2: Concurrent Respiratory & Renal Failure – Related Age-Adjusted Mortality Rates per 100,000, Stratified by States in the United States, 1999 to 2024**

| **State** | **1999–2019 AAMR (95% CI)** | **2020–2022 AAMR (95% CI)** | **2023–2024 AAMR (95% CI)** |
| --- | --- | --- | --- |
| Oregon | 4.49 (4.35–4.63) | 11.89 (11.35–12.44) | 13.52 (12.85–14.24) |
| Montana | 4.52 (4.25–4.78) | 24.07 (22.60–25.63) | 16.36 (14.90–17.95) |
| Wisconsin | 4.55 (4.44–4.67) | 13.13 (12.66–13.62) | 14.13 (13.55–14.75) |
| Wyoming | 4.60 (4.21–4.99) | 14.43 (12.82–16.21) | 16.66 (14.62–18.95) |
| Vermont | 4.70 (4.36–5.04) | 7.36 (6.39–8.50) | 7.39 (6.21–8.83) |
| Minnesota | 4.77 (4.64–4.89) | 13.03 (12.54–13.54) | 12.60 (12.03–13.20) |
| Alaska | 4.77 (4.31–5.22) | 10.41 (9.01–11.98) | 9.70 (8.11–11.56) |
| Idaho | 5.11 (4.86–5.35) | 13.74 (12.85–14.69) | 10.40 (9.49–11.39) |
| Maine | 5.26 (5.02–5.50) | 4.49 (3.97–5.09) | 4.61 (3.96–5.37) |
| New York | 5.49 (5.43–5.56) | 9.06 (8.85–9.28) | 7.58 (7.34–7.82) |
| Colorado | 5.66 (5.51–5.81) | 19.73 (19.10–20.39) | 18.83 (18.08–19.59) |
| New Mexico | 5.85 (5.63–6.08) | 14.78 (13.91–15.69) | 10.93 (10.04–11.89) |
| Iowa | 5.96 (5.79–6.13) | 17.37 (16.63–18.14) | 16.12 (15.27–17.01) |
| South Dakota | 6.11 (5.77–6.46) | 21.03 (19.44–22.74) | 18.64 (16.88–20.56) |
| Utah | 6.22 (5.98–6.46) | 13.86 (13.08–14.69) | 12.67 (11.76–13.62) |
| Maryland | 6.28 (6.14–6.42) | 13.68 (13.19–14.18) | 14.04 (13.45–14.65) |
| Louisiana | 6.49 (6.33–6.65) | 13.61 (13.05–14.20) | 11.24 (10.62–11.89) |
| Illinois | 6.53 (6.43–6.62) | 10.13 (9.84–10.42) | 9.11 (8.78–9.45) |
| Michigan | 6.60 (6.50–6.71) | 13.32 (12.96–13.70) | 11.92 (11.51–12.35) |
| Virginia | 6.73 (6.61–6.86) | 12.82 (12.42–13.23) | 11.10 (10.66–11.56) |
| Florida | 6.75 (6.68–6.82) | 12.86 (12.63–13.09) | 11.61 (11.35–11.87) |
| Arizona | 6.89 (6.75–7.02) | 14.97 (14.52–15.44) | 11.65 (11.17–12.14) |
| Massachusetts | 7.11 (6.98–7.24) | 14.16 (13.71–14.62) | 14.02 (13.48–14.57) |
| New Hampshire | 7.19 (6.89–7.49) | 10.88 (10.02–11.81) | 10.89 (9.89–12.00) |
| Washington | 7.48 (7.34–7.63) | 16.46 (15.98–16.96) | 17.26 (16.67–17.87) |
| Kansas | 7.49 (7.28–7.70) | 17.88 (17.08–18.71) | 15.49 (14.60–16.43) |
| Oklahoma | 7.84 (7.65–8.03) | 15.73 (15.08–16.42) | 12.41 (11.70–13.15) |
| North Carolina | 8.11 (7.98–8.23) | 15.93 (15.53–16.34) | 10.75 (10.35–11.15) |
| Rhode Island | 8.21 (7.87–8.55) | 9.32 (8.44–10.31) | 5.59 (4.79–6.53) |
| Connecticut | 8.23 (8.04–8.42) | 10.63 (10.10–11.18) | 6.08 (5.60–6.61) |
| Missouri | 8.25 (8.10–8.40) | 17.70 (17.16–18.26) | 16.04 (15.42–16.68) |
| North Dakota | 8.27 (7.83–8.70) | 18.58 (16.99–20.30) | 15.64 (13.89–17.58) |
| Pennsylvania | 8.29 (8.20–8.39) | 16.71 (16.36–17.06) | 16.28 (15.87–16.70) |
| New Jersey | 8.83 (8.70–8.96) | 16.97 (16.54–17.42) | 15.67 (15.17–16.18) |
| Nebraska | 8.91 (8.63–9.20) | 20.03 (18.98–21.12) | 20.53 (19.26–21.87) |
| Hawaii | 9.04 (8.71–9.36) | 9.24 (8.46–10.07) | 8.94 (8.04–9.93) |
| Georgia | 9.20 (9.05–9.34) | 16.61 (16.19–17.05) | 15.38 (14.89–15.88) |
| Ohio | 9.43 (9.31–9.54) | 16.02 (15.65–16.40) | 16.03 (15.58–16.49) |
| Tennessee | 9.51 (9.35–9.68) | 18.01 (17.38–18.66) | 18.01 (17.38–18.66) |
| Alabama | 9.93 (9.74–10.12) | 20.22 (19.58–20.89) | 18.30 (17.56–19.06) |
| Indiana | 9.94 (9.78–10.11) | 21.99 (21.40–22.60) | 15.18 (14.60–15.79) |
| Arkansas | 9.96 (9.73–10.20) | 24.06 (23.16–25.00) | 23.37 (22.30–24.48) |
| Nevada | 10.24 (9.96–10.52) | 15.28 (14.54–16.04) | 14.76 (13.90–15.66) |
| District of Columbia | 10.30 (9.73–10.87) | 11.18 (9.76–12.77) | 11.99 (10.20–14.03) |
| Kentucky | 10.73 (10.52–10.94) | 30.12 (29.10–31.16) | 30.12 (29.10–31.16) |
| California | 10.88 (10.81–10.96) | 19.72 (19.48–19.96) | 17.05 (16.78–17.32) |
| West Virginia | 11.33 (11.02–11.63) | 18.57 (17.60–19.61) | 11.81 (10.87–12.83) |
| South Carolina | 11.50 (11.29–11.71) | 17.58 (16.88–18.30) | 17.58 (16.88–18.30) |
| Mississippi | 11.86 (11.59–12.12) | 22.10 (21.02–23.23) | 22.10 (21.02–23.23) |
| Texas | 12.10 (11.99–12.20) | 23.21 (22.89–23.53) | 17.73 (17.40–18.07) |

**Supplemental Table 3: Concurrent Respiratory & Renal Failure – Related Age-Adjusted Mortality Rates per 100,000, Stratified by Census Region in the United States, 1999 to 2024**

| **Year** | **Northeast AAMR (95% CI)** | **Midwest AAMR (95% CI)** | **South AAMR (95% CI)** | **West AAMR (95% CI)** |
| --- | --- | --- | --- | --- |
| 1999 | 5.54 (5.35–5.73) | 5.13 (4.95–5.30) | 5.96 (5.80–6.11) | 6.33 (6.12–6.54) |
| 2000 | 5.92 (5.72–6.11) | 5.16 (4.98–5.33) | 6.24 (6.09–6.40) | 6.36 (6.15–6.57) |
| 2001 | 6.31 (6.11–6.51) | 5.47 (5.29–5.65) | 6.58 (6.42–6.74) | 6.81 (6.60–7.03) |
| 2002 | 6.41 (6.20–6.61) | 6.02 (5.83–6.21) | 6.76 (6.60–6.92) | 6.97 (6.75–7.19) |
| 2003 | 6.66 (6.45–6.86) | 5.84 (5.65–6.02) | 7.11 (6.95–7.27) | 7.37 (7.15–7.59) |
| 2004 | 6.89 (6.68–7.10) | 6.19 (6.01–6.38) | 7.13 (6.97–7.29) | 7.64 (7.42–7.86) |
| 2005 | 6.87 (6.66–7.07) | 6.66 (6.46–6.85) | 7.73 (7.56–7.89) | 7.84 (7.62–8.06) |
| 2006 | 6.97 (6.76–7.18) | 6.49 (6.30–6.68) | 7.69 (7.52–7.85) | 8.06 (7.84–8.29) |
| 2007 | 7.07 (6.87–7.28) | 6.76 (6.57–6.95) | 8.18 (8.01–8.35) | 8.29 (8.07–8.51) |
| 2008 | 7.31 (7.10–7.52) | 7.12 (6.93–7.32) | 8.45 (8.28–8.62) | 8.43 (8.21–8.65) |
| 2009 | 7.15 (6.94–7.35) | 7.00 (6.81–7.20) | 8.97 (8.80–9.14) | 8.86 (8.64–9.09) |
| 2010 | 7.18 (6.97–7.38) | 7.23 (7.03–7.42) | 9.39 (9.22–9.57) | 8.69 (8.47–8.91) |
| 2011 | 8.62 (8.39–8.85) | 8.84 (8.63–9.05) | 10.41 (10.23–10.59) | 10.69 (10.45–10.93) |
| 2012 | 8.32 (8.10–8.54) | 8.47 (8.26–8.68) | 10.77 (10.59–10.95) | 10.70 (10.46–10.94) |
| 2013 | 7.19 (6.98–7.39) | 7.55 (7.36–7.74) | 9.50 (9.33–9.67) | 8.61 (8.40–8.82) |
| 2014 | 7.19 (6.98–7.39) | 7.89 (7.69–8.08) | 9.71 (9.54–9.88) | 8.94 (8.73–9.15) |
| 2015 | 7.50 (7.30–7.71) | 8.36 (8.16–8.57) | 10.11 (9.94–10.28) | 9.24 (9.03–9.45) |
| 2016 | 7.14 (6.94–7.34) | 8.43 (8.23–8.63) | 10.41 (10.24–10.58) | 9.77 (9.55–9.98) |
| 2017 | 7.56 (7.36–7.76) | 8.68 (8.48–8.88) | 10.93 (10.76–11.11) | 10.35 (10.13–10.57) |
| 2018 | 7.57 (7.37–7.77) | 9.10 (8.89–9.30) | 11.36 (11.18–11.53) | 10.22 (10.01–10.43) |
| 2019 | 7.81 (7.61–8.01) | 9.21 (9.00–9.41) | 10.39 (10.23–10.56) | 9.97 (9.76–10.18) |
| 2020 | 11.45 (11.20–11.69) | 12.77 (12.52–13.01) | 14.71 (14.52–14.90) | 13.99 (13.75–14.23) |
| 2021 | 13.45 (13.19–13.72) | 16.50 (16.22–16.78) | 21.39 (21.16–21.63) | 20.40 (20.10–20.70) |
| 2022 | 13.21 (12.96–13.48) | 16.05 (15.78–16.32) | 18.36 (18.14–18.57) | 17.98 (17.70–18.25) |
| 2023 | 11.71 (11.47–11.96) | 13.91 (13.66–14.16) | 15.54 (15.34–15.74) | 15.84 (15.59–16.10) |
| 2024 | 11.28 (11.05–11.52) | 13.63 (13.38–13.87) | 14.74 (14.55–14.93) | 15.25 (15.00–15.50) |

**Supplemental Table 4: Concurrent Respiratory & Renal Failure – Related Age-Adjusted Mortality Rates per 100,000, Stratified by Urban-Rural Classification in the United States, 1999 to 2024**

| **Year** | **Metropolitan** | **Nonmetropolitan** |
| --- | --- | --- |
| **1999** | 5.90 (5.80 - 6.00) | 5.11 (4.91 - 5.31) |
| **2000** | 6.10 (5.99 - 6.20) | 5.27 (5.08 - 5.47) |
| **2001** | 6.48 (6.37 - 6.58) | 5.65 (5.44 - 5.85) |
| **2002** | 6.75 (6.65 - 6.86) | 5.79 (5.58 - 5.99) |
| **2003** | 6.91 (6.81 - 7.02) | 6.07 (5.86 - 6.28) |
| **2004** | 7.19 (7.08 - 7.30) | 6.06 (5.86 - 6.27) |
| **2005** | 7.48 (7.37 - 7.59) | 6.77 (6.55 - 6.98) |
| **2006** | 7.50 (7.40 - 7.61) | 6.78 (6.56 - 6.99) |
| **2007** | 7.80 (7.69 - 7.91) | 7.00 (6.78 - 7.22) |
| **2008** | 8.02 (7.91 - 8.13) | 7.62 (7.39 - 7.85) |
| **2009** | 8.24 (8.13 - 8.34) | 7.73 (7.50 - 7.96) |
| **2010** | 8.40 (8.29 - 8.51) | 7.99 (7.76 - 8.22) |
| **2011** | 9.82 (9.70 - 9.94) | 9.73 (9.48 - 9.99) |
| **2012** | 9.78 (9.66 - 9.89) | 9.84 (9.59 - 10.09) |
| **2013** | 8.40 (8.29 - 8.50) | 8.57 (8.33 - 8.81) |
| **2014** | 8.54 (8.44 - 8.65) | 9.31 (9.06 - 9.56) |
| **2015** | 8.90 (8.80 - 9.01) | 9.84 (9.59 - 10.09) |
| **2016** | 9.10 (8.99 - 9.20) | 10.01 (9.76 - 10.27) |
| **2017** | 9.54 (9.43 - 9.65) | 10.61 (10.35 - 10.87) |
| **2018** | 9.76 (9.65 - 9.86) | 10.91 (10.65 - 11.17) |
| **2019** | 9.32 (9.21 - 9.42) | 10.98 (10.72 - 11.25) |
| **2020** | 13.20 (13.07 - 13.32) | 15.47 (15.16 - 15.78) |

**Supplemental Table 5: Respiratory, Renal and Heart Failure – Related Age-Adjusted Mortality Rates per 100,000, in the United States, 1999 to 2024**

| **Year** | **Respiratory Failure** | **Renal Failure** |
| --- | --- | --- |
| 1999 | 58.14 (57.85–58.43) | 67.50 (67.19–67.81) |
| 2000 | 57.30 (57.02–57.58) | 70.24 (69.92–70.55) |
| 2001 | 57.50 (57.21–57.78) | 71.86 (71.55–72.17) |
| 2002 | 57.82 (57.54–58.10) | 72.94 (72.63–73.26) |
| 2003 | 57.04 (56.77–57.32) | 73.58 (73.27–73.89) |
| 2004 | 56.43 (56.16–56.70) | 72.69 (72.38–73.00) |
| 2005 | 58.33 (58.06–58.60) | 73.05 (72.74–73.35) |
| 2006 | 57.90 (57.63–58.17) | 70.45 (70.15–70.75) |
| 2007 | 58.06 (57.79–58.33) | 68.45 (68.16–68.74) |
| 2008 | 61.81 (61.53–62.08) | 66.15 (65.87–66.43) |
| 2009 | 61.25 (60.98–61.52) | 63.72 (63.44–63.99) |
| 2010 | 62.37 (62.10–62.65) | 62.15 (61.88–62.42) |
| 2011 | 65.30 (65.02–65.57) | 75.59 (75.30–75.89) |
| 2012 | 65.94 (65.67–66.22) | 74.15 (73.87–74.44) |
| 2013 | 68.91 (68.63–69.18) | 52.56 (52.32–52.80) |
| 2014 | 70.10 (69.82–70.37) | 51.23 (50.99–51.46) |
| 2015 | 74.37 (74.09–74.65) | 51.51 (51.27–51.74) |
| 2016 | 76.46 (76.18–76.74) | 50.52 (50.29–50.75) |
| 2017 | 80.50 (80.21–80.78) | 50.47 (50.25–50.70) |
| 2018 | 81.68 (81.39–81.96) | 50.13 (49.90–50.35) |
| 2019 | 79.61 (79.34–79.89) | 49.58 (49.36–49.80) |
| 2020 | 114.38 (114.05–114.71) | 58.82 (58.58–59.06) |
| 2021 | 133.16 (132.80–133.53) | 69.73 (69.47–70.00) |
| 2022 | 105.95 (105.64–106.27) | 65.98 (65.73–66.23) |
| 2023 | 93.21 (92.91–93.50) | 59.74 (59.51–59.98) |
| 2024 | 90.18 (89.89–90.46) | 57.59 (57.36–57.82) |

**Supplemental Table 6: Concurrent Respiratory & Renal Failure - Related Age-Adjusted Mortality Rates per 100,000 Stratified by Renal Failure Subtype (Acute vs Chronic Renal Failure) in the United States, 1999–2024**

| **Year** | **Acute Renal Failure + Respiratory Failure AAMR (95% CI)** | **Chronic Renal Failure + Respiratory Failure AAMR (95% CI)** |
| --- | --- | --- |
| 1999 | 1.16 (1.12–1.20) | 1.15 (1.11–1.19) |
| 2000 | 1.20 (1.16–1.24) | 1.26 (1.22–1.31) |
| 2001 | 1.32 (1.28–1.36) | 1.38 (1.34–1.43) |
| 2002 | 1.44 (1.40–1.49) | 1.49 (1.44–1.53) |
| 2003 | 1.57 (1.53–1.62) | 1.57 (1.53–1.62) |
| 2004 | 1.73 (1.69–1.78) | 1.65 (1.60–1.70) |
| 2005 | 1.92 (1.87–1.97) | 1.74 (1.70–1.79) |
| 2006 | 2.08 (2.03–2.13) | 1.83 (1.78–1.88) |
| 2007 | 2.28 (2.22–2.33) | 1.89 (1.84–1.94) |
| 2008 | 2.71 (2.66–2.77) | 1.92 (1.87–1.97) |
| 2009 | 3.04 (2.98–3.10) | 2.03 (1.98–2.08) |
| 2010 | 3.37 (3.31–3.43) | 2.03 (1.98–2.08) |
| 2011 | 3.51 (3.44–3.57) | 3.60 (3.53–3.66) |
| 2012 | 3.50 (3.43–3.56) | 3.82 (3.75–3.88) |
| 2013 | 3.74 (3.67–3.80) | 2.80 (2.74–2.85) |
| 2014 | 3.93 (3.86–3.99) | 3.00 (2.94–3.06) |
| 2015 | 4.06 (4.00–4.13) | 3.38 (3.32–3.44) |
| 2016 | 4.25 (4.19–4.32) | 3.70 (3.64–3.76) |
| 2017 | 4.41 (4.34–4.48) | 4.09 (4.02–4.15) |
| 2018 | 4.49 (4.43–4.56) | 4.30 (4.23–4.36) |
| 2019 | 4.24 (4.17–4.30) | 4.29 (4.23–4.36) |
| 2020 | 6.59 (6.51–6.67) | 5.80 (5.73–5.87) |
| 2021 | 11.21 (11.10–11.31) | 6.39 (6.32–6.47) |
| 2022 | 10.65 (10.55–10.75) | 5.58 (5.51–5.65) |
| 2023 | 8.93 (8.84–9.02) | 5.15 (5.08–5.22) |
| 2024 | 8.41 (8.32–8.50) | 5.06 (4.99–5.13) |

**Supplemental Table 7: Concurrent Respiratory & Renal Failure - Related Age-Adjusted Mortality Rates per 100,000 Stratified by Respiratory Failure Subtype (Acute vs Chronic Respiratory Failure) in the United States, 1999–2024**

| **Year** | **Acute Respiratory Failure + Renal Failure, AAMR (95% CI)** | **Chronic Respiratory Failure + Renal Failure, AAMR (95% CI)** |
| --- | --- | --- |
| 1999 | 0.62 (0.59–0.65) | 0.04 (0.03–0.05) |
| 2000 | 0.66 (0.63–0.69) | 0.07 (0.06–0.08) |
| 2001 | 0.65 (0.62–0.68) | 0.08 (0.07–0.09) |
| 2002 | 0.71 (0.68–0.75) | 0.10 (0.09–0.11) |
| 2003 | 0.75 (0.72–0.78) | 0.12 (0.11–0.14) |
| 2004 | 0.83 (0.80–0.87) | 0.13 (0.12–0.14) |
| 2005 | 0.91 (0.87–0.94) | 0.15 (0.14–0.17) |
| 2006 | 1.01 (0.97–1.04) | 0.16 (0.14–0.17) |
| 2007 | 1.08 (1.05–1.12) | 0.19 (0.17–0.20) |
| 2008 | 1.38 (1.34–1.42) | 0.24 (0.22–0.26) |
| 2009 | 1.68 (1.64–1.73) | 0.29 (0.27–0.31) |
| 2010 | 2.01 (1.96–2.06) | 0.30 (0.28–0.32) |
| 2011 | 2.66 (2.60–2.71) | 0.45 (0.43–0.47) |
| 2012 | 2.88 (2.82–2.93) | 0.55 (0.52–0.57) |
| 2013 | 2.97 (2.91–3.03) | 0.47 (0.44–0.49) |
| 2014 | 3.41 (3.35–3.47) | 0.56 (0.54–0.59) |
| 2015 | 3.97 (3.91–4.04) | 0.71 (0.68–0.73) |
| 2016 | 4.43 (4.36–4.50) | 0.78 (0.75–0.80) |
| 2017 | 4.76 (4.69–4.83) | 0.88 (0.85–0.91) |
| 2018 | 4.91 (4.84–4.98) | 0.97 (0.94–1.00) |
| 2019 | 4.80 (4.73–4.87) | 1.01 (0.98–1.04) |
| 2020 | 7.62 (7.54–7.71) | 1.15 (1.11–1.18) |
| 2021 | 11.05 (10.94–11.15) | 1.46 (1.42–1.50) |
| 2022 | 11.04 (10.93–11.14) | 1.58 (1.55–1.62) |
| 2023 | 9.44 (9.35–9.53) | 1.62 (1.58–1.66) |
| 2024 | 9.17 (9.08–9.26) | 1.55 (1.51–1.58) |

**Supplemental Table 8: Distribution of Underlying Cause of Death Among Decedents With Concurrent Respiratory and Renal Failure, Stratified by Renal and Respiratory Phenotypes, in the United States**

| **Cohort / Phenotype** | **Total Deaths, n** | **UCOD: Renal Failure, n (%)** | **UCOD: Respiratory Failure, n (%)** | **UCOD: Other Causes, n (%)** |
| --- | --- | --- | --- | --- |
| **Pre-pandemic (1999-2019)** |  |  |  |  |
| **Overall Concurrent Respiratory + Renal Failure** | 569,159 | 117,483 (20.64) | 4,359 (0.77) | 447,317 (78.59) |
| **Renal Phenotype Stratification** |  |  |  |  |
| Respiratory Failure + Acute Kidney Failure (N17) | 207,622 | 37,761 (18.19) | 1,301 (0.63) | 168,560 (81.18) |
| Respiratory Failure + Chronic Kidney Disease (N18) | 184,056 | 46,949 (25.51) | 2,204 (1.20) | 134,903 (73.29) |
| Respiratory Failure+ Unspecified Renal Failure | 198,074 | - | - | - |
| **Respiratory Phenotype Stratification** |  |  |  |  |
| Renal Failure + Acute Respiratory Failure (J96.0) | 200,058 | 33,731 (20.00) | 1,379 (0.82) | 164,948 (80.20) |
| Renal Failure + Chronic Respiratory Failure (J96.1) | 30,280 | 4,372 (14.44) | 2,664 (8.60) | 23,244 (76.96) |
| Renal Failure+ Respiratory Failure Unspecified | 387,215 | - | - | - |
| **Pandemic period (2020-2022)** |  |  |  |  |
| Overall Concurrent Respiratory + Renal Failure | 569,159 | 28,709 (14.19) | 1,510 (0.75) | U00-U49 (Provisional assignment of new diseases of uncertain etiology) 55,225 (27.30) Others 483,715 (84.98) |
| **Post pandemic period (2023-2024)** |  |  |  |  |
| Overall Concurrent Respiratory + Renal Failure | 122,820 | 20,539 (16.72) | 1,273 (1.04) | J40-J47 (Chronic lower respiratory diseases) 6957(5.66)  K70-K76 (Diseases of liver) 5611 (4.57)  J09-J18 (Influenza and pneumonia) 5417 (4.41)  U00-U49 (Provisional assignment of new diseases of uncertain etiology) 5,020 (4.09)  I20-I25 (Ischaemic heart diseases) 7,416(6.04)  I30-I51 (Other forms of heart disease) 10,606 (8.64)  C00-C97 (Malignant neoplasms) 14,162(11.53) Rest others |

***Note: Subgroup totals exceed the overall cohort total (N=625,051) because ICD-10 coding on death certificates is not mutually exclusive. Decedents may have multiple overlapping codes (e.g., acute on chronic renal failure) and are therefore represented in multiple phenotypic strata.**

**Supplementary Methods: CDC WONDER Query Settings**

To ensure reproducibility, the exact query parameters used to generate the primary dataset for this study are listed below. These settings can be applied in the CDC WONDER Multiple Cause of Death (1999–2020), (2018-2024) online database.

**Section 1**

Group Results By: Year, sex, race, census region, urbanization, states, age, place of death

Measures: Deaths, Population, Crude Rate, Age-Adjusted Rate, 95% Confidence Interval, Standard Error.

Rates were calculated by 100,000 population

Age-Adjustment: 2000 U.S. Standard Population

**Section 2**

States: All States, 2013 urbanization, All categories

**Section 3**

Age Group: All Ages, Gender: All, Race: All, Hispanic Origin: All

**Section 4**

Year/Month: All Years (1999–2020), (2021-2024)

**Section 5:** All

**Section 6**: UCD - ICD-10 Codes: All (Default)

**Section 7:** MCD - ICD-10 Codes

Group A (Respiratory Failure): J96 (Respiratory failure, not elsewhere classified)

Group B (Renal Failure): N17 (Acute kidney failure), N18 (Chronic kidney disease), N19 (Unspecified kidney failure)

Logic: The query was constructed to select records containing Any code from Group A AND Any code from Group B.

**Section 8**

Precision: 2 decimal place
